# Living Alone Transitions and Mortality in Older Men and Women

**DOI:** 10.1093/geroni/igaa057.2205

**Published:** 2020-12-16

**Authors:** Jessica Abell, Andrew Steptoe

**Affiliations:** University College London, London, England, United Kingdom

## Abstract

Living alone has been established as a risk factor for mortality, with biopsychosocial mechanisms suggested as plausible. However, it is unclear whether this is due to health selection. We analysed data from 4,888 individuals who participated in both wave 2 (2004-2005) and wave 4 (2008-2009) of the English Longitudinal Study of Ageing. Mortality status was ascertained from linked mortality register data. An association was found between living alone at wave four and mortality (HR: 1·20, 95% CI 1·04–1·38) in a model adjusted for a range of factors. We also found that participants who transitioned into a solo household due to divorce or bereavement had a higher risk of mortality (HR: 1·34, 95% CI 1·01–1·79). Transitioning into a solo household is also associated with mortality and the underlying reason for this transition was found to be important.

